# Predictors of Recovery Following Lumbar Microdiscectomy for Sciatica: A Systematic Review and Meta-Analysis of Observational Studies

**DOI:** 10.7759/cureus.39664

**Published:** 2023-05-29

**Authors:** Yasir Rehman, Malgorzata Bala, Nadia Rehman, Arnav Agarwal, Magdalena Koperny, Holly Crandon, Ream Abdullah, Alexandra Hull, Nima Makhdami, Savannah Grodecki, Anna Wrzosek, Wiktoria Lesniak, Nathan Evaniew, Vahid Ashoorion, Li Wang, Rachel Couban, Brian Drew, Jason W Busse

**Affiliations:** 1 Health Research Methodology, McMaster University, Hamilton, CAN; 2 Epidemiology and Preventive Medicine, Jagiellonian University Medical College, Krakow, POL; 3 Health Research Methods, Impact and Evidence, McMaster University, Hamilton, CAN; 4 Medicine, University of Toronto, Toronto, CAN; 5 Michael G. DeGroote Institute for Pain Research and Care, McMaster University, Hamilton, CAN; 6 Medicine, McMaster University, Hamilton, CAN; 7 Family Medicine, University of British Columbia, Vancouver, CAN; 8 Interdisciplinary Intensive Care, Jagiellonian University, Krakow, POL; 9 Medicine, Jagiellonian University Medical College, Krakow, POL; 10 Surgery, University of Calgary, Calgary, CAN; 11 Neurosurgery, McMaster University, Hamilton, CAN

**Keywords:** persistent post-surgical leg pain, postoperative functional impairment, lumbar disc herniation surgery, systematic review and meta-analysis, prognosis, return to work, lumbar microdiscectomy

## Abstract

Chronic post-surgical pain is reported by up to 40% of patients after lumbar microdiscectomy for sciatica, a complaint associated with disability and loss of productivity.

We conducted a systematic review of observational studies to explore factors associated with persistent leg pain and impairments after microdiscectomy for sciatica. We searched eligible studies in MEDLINE, Embase, and CINAHL that explored, in an adjusted model, predictors of persistent leg pain, physical impairment, or failure to return to work after microdiscectomy for sciatica. When possible, we pooled estimates of association using random-effects models using the Grading of Recommendations Assessment, Development, and Evaluation approach.

Moderate-certainty evidence showed that the female sex probably has a small association with persistent post-surgical leg pain (odds ratio (OR) = 1.15, 95% confidence interval (CI) = 0.63 to 2.08; absolute risk increase (ARI) = 1.8%, 95% CI = -4.7% to 11.3%), large association with failure to return to work (OR = 2.79, 95% CI = 1.27 to 6.17; ARI = 10.6%, 95% CI = 1.8% to 25.2%), and older age is probably associated with greater postoperative disability (β = 1.47 points on the 100-point Oswestry Disability Index for every 10-year increase from age (>/=18 years), 95% CI = -4.14 to 7.28). Among factors that were not possible to pool, two factors showed promise for future study, namely, legal representation and preoperative opioid use, which showed large associations with worse outcomes after surgery.

The moderate-certainty evidence showed female sex is probably associated with persistent leg pain and failure to return to work and that older age is probably associated with greater post-surgical impairment after a microdiscectomy. Future research should explore the association between legal representation and preoperative opioid use with persistent pain and impairment after microdiscectomy for sciatica.

## Introduction and background

The lifetime prevalence of sciatica in the general population ranges from 12% to 43% [1,2] and is associated with pain radiating down the leg, numbness, and motor deficits [1,3]. In the United States, the total direct and indirect costs (e.g., loss of wages and productivity) associated with sciatica exceed $50 billion annually [4-6]. Lumbar discectomy is an elective surgical procedure performed in approximately 10% of sciatica patients to relieve symptoms and promote functional recovery [7-9]; however, outcomes are variable, and up to 40% of patients report persistent post-surgical leg pain [10-12].

Previous systematic reviews have identified greater preoperative pain severity, comorbid mental illness, receipt of worker’s compensation benefits, and higher fear avoidance as risk factors for poor outcomes following surgical decompression for sciatica [11,13-16]. However, prior reviews have several limitations, such as outdated searches [17], language restrictions [13,15,17], and the inclusion of studies reporting predictors from unadjusted analyses [14,16,17]. We conducted a systematic review of observational studies to identify risk factors for persistent leg pain and impairment following microdiscectomy for sciatica that addresses the limitations of prior reviews. A greater understanding of factors associated with poor outcomes following decompression for sciatica may further optimize decision-making between patients and their surgeons [18].

## Review

Methodology

We completed our review in accordance with the MOOSE [19] and Preferred Reporting Items for Systematic Reviews and Meta-Analyses (PRISMA) 2020 statements [20] and registered our protocol with PROSPERO (CRD42015019526).

Literature Search

We searched Medline, EMBASE, and CINAHL, without language restrictions, from inception to August 2021 (details of our literature search strategy and the search terms used are provided in the Appendices). We reviewed bibliographic references of all eligible studies as well as six previous systematic reviews [13-17,21] for additional eligible articles.

We included cohort or case-control studies that explored, in an adjusted analysis, risk factors for persistent pain, disability, or unemployment after lumbar microdiscectomy for sciatica. We excluded randomized controlled trials (RCT) from our review as RCTs follow strict recruitment criteria, which mask the important prognostic factors that can be explored in observational studies. Second, the application of intervention in RCTs might confound the true association of exposure variables with the outcome variables.

Studies enrolling patients with spinal stenosis or spondylolisthesis, or who underwent fusion in addition to microdiscectomy, or repeat spine surgery were not eligible for review. Eligible procedures included microdiscectomy, endoscopic microdiscectomy, microendoscopic discectomy, mini-open discectomy, and tubular microdiscectomy. We excluded randomized trials as strict eligibility criteria may exclude patients with important prognostic factors. We also excluded non-randomized studies with interventions, descriptive or qualitative studies, and letters to the editors. We excluded studies that reported only adjusted models with significant association with variables collected after baseline, as in such instances the direction of association is uncertain.

Study Selection and Data Abstraction

Trained reviewers worked in pairs to screen titles and abstracts of identified citations and full texts of all potentially eligible studies independently and in duplicate. All reviewers completed pilot exercises before screening to increase reliability. Disagreements were resolved through discussion or, when necessary, by an arbitrator (JWB).

The same pairs of reviewers independently extracted data from eligible articles, including sample size, duration of follow-up, patient characteristics, and measures of association for all factors assessed for an association with persistent leg pain, functional disability, or return to work (RTW) following lumbar discectomy. If a study reported multiple follow-up times, we captured data for the longest follow-up reported.

Risk of Bias

We used criteria from Users’ Guides to the Medical Literature [22] to assess the risk of bias: (1) representativeness of the study population (low risk of bias when using random sampling, consecutive sampling, or data collected from a patient registry; high risk of bias when the source of the study population was not reported or acquired through convenience sampling); (2) validity of outcome assessment; (3) loss to follow-up (>20% was considered high risk of bias); and (4) whether predictive models were optimally adjusted (low risk of bias if adjusted, at minimum, for age, sex, and baseline pain severity).

Data Analysis

We assessed the reliability of full-text screening with the kappa statistic [23]. When possible, we pooled all factors assessed for an association with persistent pain, disability, or unemployment and reported by at least two studies. For categorical variables, we reported pooled estimates as odds ratios (ORs) and associated 95% confidence intervals (95% CIs), and for continuous variables, we reported pooled estimates as beta coefficients (β) and associated 95% CIs using DerSimonian-Laird random-effects models. To avoid overestimating the strength of association by restricting pooling to risk factors with reported associations, we imputed an OR of 1 and an associated measure of precision using the hot deck approach [24,25] for all categorical predictors that were reported as non-significant and without accompanying data. We complimented ORs with the absolute risk increase for each predictor amenable to meta-analysis. We acquired the following baseline risks from the low-risk group in the study with the largest sample size among studies eligible for our review at low risk of bias: (1) 14% for persistent post-surgical leg pain [26], (2) 20% for persistent disability [27], and (3) 7% for failure to RTW [28]. We used SPSS Statistics version 28.01.1.0 (IBM Corp., Armonk, NY, USA) for all statistical analyses; all comparisons were twp-tailed, and p-values ≤0.05 were considered statistically significant.

When pooling was not possible, we explored the consistency of the association between pooled results and studies reporting the same predictors that could not be pooled. We used the following three criteria to identify predictors that were not amenable to pooling and showed promise for future research: (1) a statistically significant association of p ≤ 0.01, (2) a large magnitude of association (OR ≥2.0 or <0.5), and (3) a sample size of ≥500.

Subgroup Analyses

We evaluated heterogeneity for all pooled estimates through visual inspection of forest plots. We generated three hypotheses to explore heterogeneity between studies, assuming larger associations with (1) a higher risk of bias on a criterion-by-criterion basis, (2) a longer duration of follow-up, and (3) a higher threshold for outcomes (e.g., moderate-to-severe persistent leg pain vs. any persistent leg pain). We only conducted subgroup analysis if there were at least two studies in each subgroup and assessed the credibility of significant subgroup effects using the modified ICEMAN criteria [29].

Sensitivity Analysis

We performed a sensitivity analysis by removing imputed data from our pooled analyses.

Certainty of Evidence

We used the Grading of Recommendations Assessment, Development, and Evaluation (GRADE) approach to summarize the certainty of evidence for all meta-analyses [30]. With this approach, the evidence for prognostic factors begins as high certainty but can be downgraded to moderate, low, or very low based on the risk of bias, consistency, directness, precision, and publication bias [31]. Accordingly, while associations supported by high certainty evidence are presented without any qualifiers, moderate certainty evidence is preceded with the qualifier “probably” and low certainty evidence with the qualifier “may.” If subgroup analysis for risk of bias did not find a significant association, we included all studies and did not rate down for risk of bias. If we found a credible subgroup effect for risk of bias, we pooled only low-risk studies [32].

On a review of baseline risks for our outcomes, clinical experts from our team (BD, NE) estimated that a 5% increase in absolute risk would be sufficient for clinicians to address modifiable risk factors, and an absolute difference in risk of 10% between groups at low and high risk for persistent pain, prolonged disability, or unemployment would be sufficient for clinicians to selectively target non-modifiable risk factors. Therefore, we rated down for imprecision if the 95% CI associated with the risk difference included 5% for modifiable risk factors or 10% for non-modifiable risk factors. For meta-analyses with at least 10 studies, we assessed publication bias by visual assessment of the asymmetry of the funnel plot and performed the Begg rank correlation test and the Egger test [33,34].

Results

**Figure 1:**
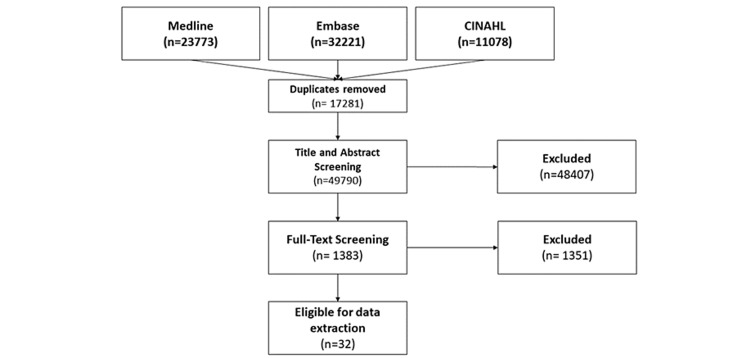
Preferred Reporting Items for Systematic Reviews and Meta-Analyses flow chart for the literature search and screening process.

Our literature search yielded 49,790 unique citations, of which 32 studies [27,35-66] with 26,876 participants were eligible for review (Figure 1). The median sample size among eligible studies was 143 (range = 40 to 14,097), and the median follow-up time after surgery was 12 months (range = 3 to 36). Only nine of 32 studies (29%) included all predictors in their final adjusted analysis [35,43,49-52,61,64,65] (Table 1).

**Table 1:** Summary characteristics of reviewed studies. RTW = return to work; ANCOVA = analysis of covariance; * = median

Author, year	Sample size in the final model	Mean age (SD)	Sex (female; %)	Follow-up duration (months)	Surgery type	All predictors included in the final analysis
Chaichana et al., 2011 [35]	67	41 (10)	37	12	Lumbar microdiscectomy	Yes
Den Boer et al., 2006 [36]	277	43 (17)	50	6	Lumbar discectomy	No
Den Boer et al., 2006 [37]	182	41	41	6	Lumbar discectomy	No
En'Wezoh et al., 2017 [38]	63	44 (12)	38	3	Lumbar microdiscectomy	No
Ford et al., 2020 [39]	94	45 (13)	31	6	Lumbar microdiscectomy	No
Hegarty et al., 2012 [40]	53	39*	47	3	Lumbar microdiscectomy	No
Hareni et al., 2021 [65]	14,097	43 (11)	45	12	Lumbar microdiscectomy	
Johansson et al., 2010 [41]	55	40 (8)	40	12	Lumbar microdiscectomy	No
Koen et al., 2017 [61]	92	52 (10)	54	12	Lumbar discectomy	Yes
Lagerbäck et al., 2019 [27]	6,468	43 (11)	43	6	Lumbar open/microdiscectomy	Yes
Laufenberg-Feldmann et al., 2018 [62]	106	59 (17)	48	6	Percutaneous endoscopic lumbar discectomy	Yes
Lee et al., 2010 [42]	40	50	43	23.5 (mean)	Lumbar discectomy	No
Mayo et al., 2017 [43]	110	41 (12)	32	6	Lumbar microdiscectomy	Yes
Moranjkic et al., 2010 [44]	70	49 (9)	49	6	Lumbar microdiscectomy	No
O’Donnell et al., 2018 [45]	1,286	40 (10)	24	36	Lumbar discectomy	No
Patel et al., 2019 [46]	188	43 (14)	37	12	Lumbar microdiscectomy	No
Quon et al., 2013 [47]	291	43 (13)	38	6	Lumbar open/microdiscectomy	No
Rut et al., 2014 [48]	176	47	42	6	Lumbar microdiscectomy	No
Schade et al., 1999 [49]	42	Not reported	Nor reported	12	Lumbar microdiscectomy	Yes
Shamji et al., 2016 [50]	250	58 (16)	44	6	Lumbar microdiscectomy	Yes
Shrestha et al., 2015 [51]	63	43 (9)	32	34.8 (mean)	Lumbar microdiscectomy	Yes
Sicolli et al., 2019 [52]	372	48 (12)	51	12	Tubular microdiscectomy	No
Solberg et al., 2005 [53]	180	41 (11)	37	12	Lumbar microdiscectomy	No
Sørlie et al., 2012 [54]	178	41 (12)	37	12	Lumbar microdiscectomy	No
Than et al., 2016 [55]	127	45, IQR = 37.0–54.0	48	12	Lumbar microdiscectomy	No
Udby et al., 2020 [66]	620	51*	50	24	Lumbar discectomy	Not clear
Voorhies et al., 2007 [57]	110	NR	NR	12	Lumbar discectomy	No
Vucetic et al., 1999 [58]	160	43 (10)	47	24	Lumbar discectomy	No
Willems et al., 2020 [63]	298	44.9 (13.1)	40.6	12	Lumbar microdiscectomy	No
Weir 1979 [59]	42	42	25	12	Lumbar discectomy	No
Ziegler et al., 2020 [60]	351	42 (10)	46	24	Lumbar open/microdiscectomy	No
Ziegler et al., 2021 [64]	835	43 (14)	48	12	Lumbar open/microdiscectomy	No

Risk of Bias

Among eligible studies, 15 [36,37,40-42,44,47,50,52-54,58,60,62,65] were at low risk of bias for all criteria. Six studies [27,51,55,59,62,64] either did not report loss to follow-up or acknowledged >20% missing data. Fourteen studies [35,38,39,43,45,46,48,49,55,57,59,63,64,66] did not adjust their final models for age, gender, or baseline pain severity (Table 2).

**Table 2:** Risk of bias analysis of the reviewed studies.

Author, year	Representativeness of the study population	Valid outcome measure	Lost to follow-up (high risk if >20%)	Adjusted factors (age, gender, and preoperative pain)	Comments
Chaichana et al., 2011 [35]	Low risk	Low risk	Low risk	High risk	Not adjusted for gender
Den Boer et al., 2006 [36]	Low risk	Low risk	Low risk	Low risk	
Den Boer et al., 2006 [37]	Low risk	Low risk	Low risk	Low risk	
En'Wezoh et al., 2017 [38]	Low risk	Low risk	Low risk	High risk	Not adjusted for preoperative pain severity
Ford et al., 2020 [39]	Low risk	Low risk	Low risk	High risk	Not adjusted for age, gender, and preoperative pain severity.
Hegarty et al., 2012 [40]	Low risk	Low risk	Low risk	Low risk	
Hareni et al., 2021 [65]	Low risk	Low risk	Low risk	Low risk	
Johansson et al., 2010 [41]	Low risk	Low risk	Low risk	Low risk	
Koen et al., 2017 [61]	Low risk	Low risk	Low risk	Low risk	
Lagerbäck et al., 2019 [27]	Low risk	Low risk	High risk	Low risk	
Laufenberg-Feldmann et al., 2018 [62]	Low risk	Low risk	High risk	Low risk	
Lee et al., 2010 [42]	Low risk	Low risk	Low risk	Low risk	
Mayo et al., 2017 [43]	Low risk	Low risk	Low risk	High risk	Not adjusted for preoperative pain severity
Moranjkic et al., 2010 [44]	Low risk	Low risk	Low risk	Low risk	
O’Donnell et al., 2018 [45]	Low risk	Low risk	Low risk	High risk	Not adjusted for preoperative pain severity
Patel et al., 2019 [46]	Low risk	Low risk	Low risk	High risk	Not adjusted for preoperative pain severity
Quon et al., 2013 [47]	Low risk	Low risk	Low risk	Low risk	
Rut et al., 2014 [48]	Low risk	Low risk	Low risk	High risk	Not adjusted for preoperative pain severity
Schade et al., 1999 [49]	Low risk	Low risk	Low risk	High risk	Not adjusted for age and gender
Shamji et al., 2016 [50]	Low risk	Low risk	Low risk	Low risk	
Shrestha et al., 2015 [51]	Low risk	Low risk	High risk	Low risk	
Sicolli et al., 2019 [52]	Low risk	Low risk	Low risk	Low risk	
Solberg et al., 2005 [53]	Low risk	Low risk	Low risk	Low risk	
Sørlie et al., 2012 [54]	Low risk	Low risk	Low risk	Low risk	
Than et al., 2016 [55]	Low risk	Low risk	High risk	High risk	Not adjusted for preoperative pain severity
Udby et al., 2020 [66]	Low risk	Low risk	Low risk	High risk	Not adjusted for gender
Voorhies et al., 2007 [57]	Low risk	Low risk	Low risk	High risk	Not adjusted for age and gender
Vucetic et al., 1999 [58]	Low risk	Low risk	Low risk	Low risk	
Willems et al., 2020 [63]	Low risk	Low risk	Low risk	High risk	Not adjusted for age and gender
Weir et al., 1979 [59]	Low risk	Low risk	High risk	High risk	Not adjusted for age and gender
Ziegler et al., 2020 [60]	Low risk	Low risk	Low risk	Low risk	
Ziegler et al., 2021 [64]	Low risk	Low risk	High risk	High risk	Not adjusted for gender

Predictors of Persistent Post-surgical Pain

**Figure 2:**
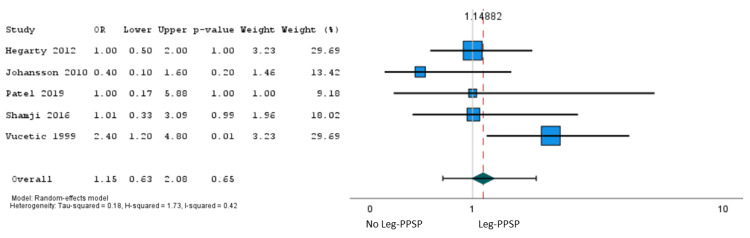
A pooled analysis of the PPSP - leg pain: female sex (reference: males). PPSP = persistent post-surgical pain

In total, 22 studies [27,35,36,39,40,41,44,46-50,52,54,57,58,59,61,62,65,66] involving 24,156 patients reported the association of 48 independent variables with persistent pain after lumbar discectomy, among which only sex met our criteria for meta-analysis. Moderate certainty evidence from five studies (n = 706) showed a small association between the female sex and persistent leg pain after lumbar microdiscectomy (OR = 1.15, 95% CI = 0.63 to 2.08) (Figure 2). The absolute risk increase in persistent leg pain associated with the female sex was 1.8% (95% CI = -4.7% to 11.3%) (Table 3).

**Table 3:** GRADE evidence profile: predictors of recovery after microdiscectomy for sciatica. * = unless otherwise indicated; ! = O’Donnell et al. [45], Than et al. [55], and Ziegler et al. [64] did not optimally adjust the final model for age, gender, and preoperative pain intensity. The test between subgroup homogeneity was significant (p = 0.03). Therefore, the quality of evidence was determined by the low risk of bias studies. Quality was rated down based on imprecision because the 95% CI associated with the risk difference included the predefined threshold of 5% for modifiable factors or 10% for non-modifiable factors, which means that clinical actions based on the estimate of the lower or upper boundary may be different. CI = confidence interval; GRADE = Grading of Recommendations, Assessment, Development, and Evaluations

Predictor (number of studies [patients])	Risk of bias	Inconsistency		Imprecision	Indirectness	Publication bias	Baseline risk	OR (95% CI)*	Risk difference (95% CI)	GRADE Rating
Persistent post-surgical pain - leg pain										
Female sex (5 [706 patients] Median follow-up (12 months)	No serious risk of bias		No serious inconsistency	Serious imprecision	No serious indirectness	Undetected; only five studies	14.3%	1.15 (0.63, 2.08)	1.8% (-4.7%, 11.3%)	Moderate
Functional disability [(Oswestry Disability Index (0–100)]										
Age, per 10-year increase from the median age of 42.52 (5 [7043 patients]) Median follow-up (24 months)	No serious risk of bias		No serious inconsistency	Serious imprecision	No serious indirectness	Undetected; only five studies	19.87%	Beta-coefficient = 1.57 (-4.14, 7.28)		Moderate
Failure to return to work										
Female sex (2 [210 patients]) Median follow-up (24 months)	No serious risk of bias		No serious inconsistency	Serious imprecision	No serious indirectness	Undetected; only two studies	7.2%	2.79 (1.27, 6.17)	10.6% (1.8%, 25.2%)	Moderate

Predictors of Functional Disability

**Figure 3:**
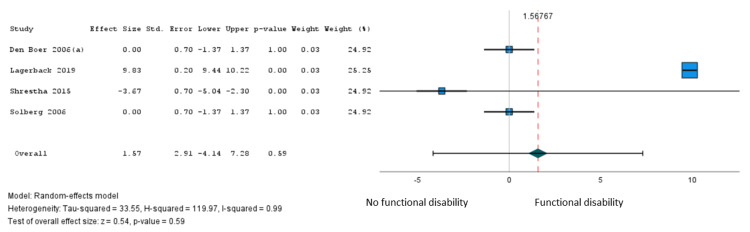
Pooled analysis of disability outcomes - age (10 years increase); Oswestry Disability Index (0-100 scale).

**Figure 4:**
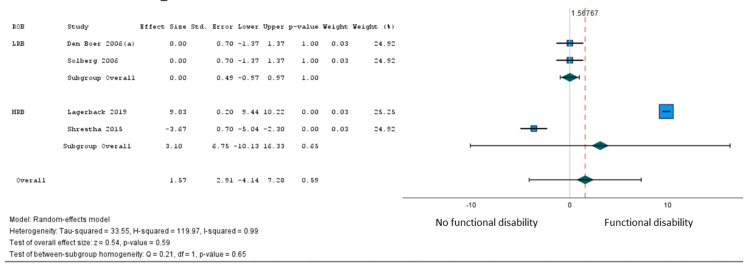
Subgroup analysis of disability outcomes age (10 years increase); Oswestry Disability Index (0-100 scale); subgroup analysis based on high risk of bias (loss of follow-up >20%).

In total, 16 studies [27,35,36,38,39,41-44,46,49,51,53,64,66] involving 9,192 patients examined the association of 41 variables with a persistent postoperative functional disability after lumbar microdiscectomy for sciatica. Only age met our criteria for pooling. Moderate certainty evidence from five studies (7,043 patients) [27,36,51,53] supported a modest association between older age and greater postoperative disability (β = 1.57 points on the 100-point Oswestry Disability Index for every 10-year increase in age from 18 years, 95% CI = -4.14 to 7.28) (Figure 3, Table 3). The subgroup analysis based on the loss of follow-up >20% was not significant (Figure 4).

Predictors of Failure to Return to Work

**Figure 5:**
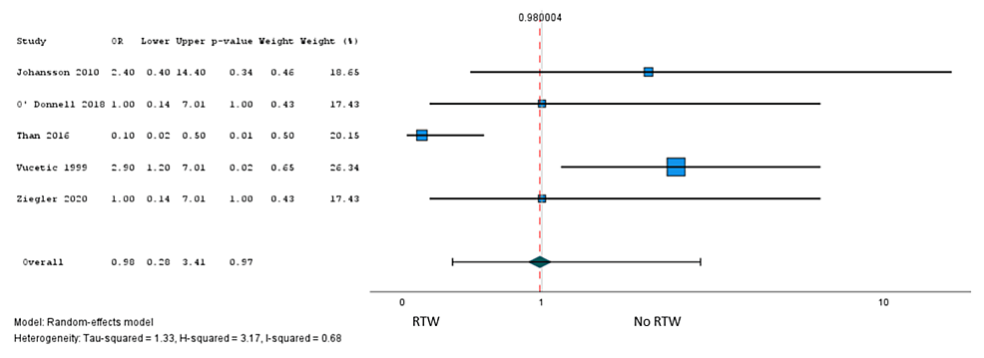
A pooled analysis of the failure RTW: female sex (reference: males). RTW = return to work

**Figure 6:**
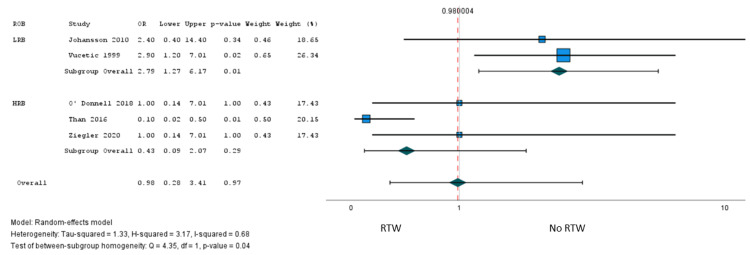
Subgroup analysis of failure RTW: female sex (reference: males). Subgroup analysis based on the risk of bias (lack of optimally adjusted model). RTW = return to work

A total of six studies [41,45,55,58,60] involving 2,021 patients reported the association of 39 factors with RTW after surgery, and one study [37] (n = 141 patients) explored the association with postoperative working capacity. Only sex met our criteria for meta-analysis, and low certainty evidence from five studies [41,45,55,58,60] (1,979 patients) suggested little to no association with failure to RTW after surgery (OR = 0.98, 95% CI = 0.28 to 3.41) (Figure 5). However, we found evidence of a credible subgroup effect based on whether studies reported an optimally adjusted predictive model (Appendices: ICEMAN criteria). We found moderate certainty evidence from two studies (210 patients) that reported optimally adjusted predictive models that the female sex versus male sex was probably associated with greater odds of failure to RTW after surgery (OR = 2.79, 95% CI = 1.27 to 6.17; risk difference = 11%, 95% CI = 2% to 25%) (Figure 6, Table 3).

Sensitivity Analysis

**Figure 7:**
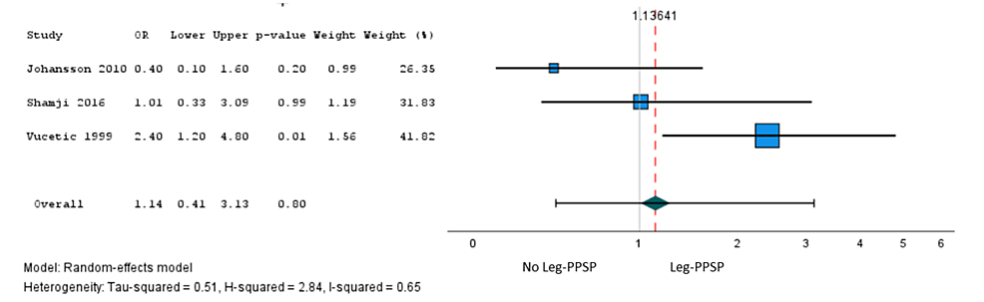
Sensitivity analysis of PPSP leg pain: female sex (reference: males). PPSP = persistent post-surgical pain

**Figure 8:**
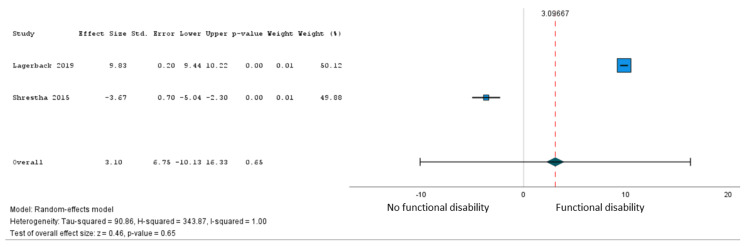
Sensitivity analysis of disability outcomes age (10 years increase); Oswestry Disability Index (0-100 scale).

**Figure 9:**
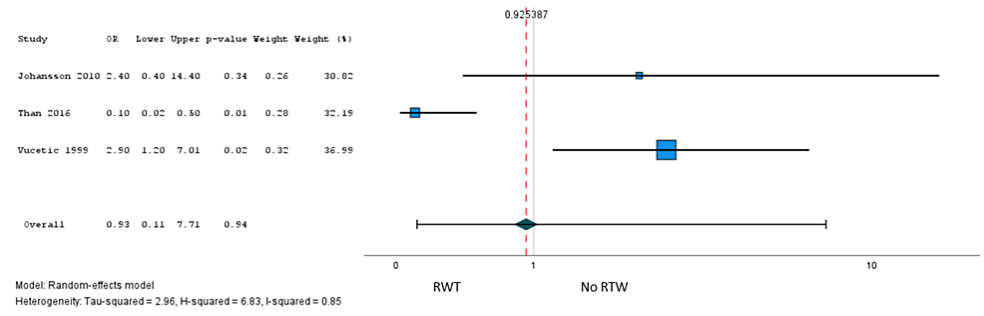
Sensitivity analysis of failure RTW: female sex (reference: males). RTW = return to work

The sensitivity analyses for the leg pain PPSP, functional impairment, and RTW are shown in Figures 7-9. The association of the female sex with leg PPSP (OR (95% CI) = 1.14 (0.41, 3.13), RTW (OR (95% CI) = 0.93 (0.11, 7.71), and age per 10 years increase β (95% CI) = 3.10 (-10.13, 16.33)).

Variables Not Amenable to Meta-Analysis

Tables 4-9 present the associations with persistent pain, persistent disability, and failure to RTW (38 variables) for approximately 50 variables factors that were not amenable to meta-analysis. Two of these factors, opioid use before surgery and legal representation at the time of surgery, met our criteria as promising for future investigations (Table 6).

**Table 4:** Factors of persistent post-surgical pain (PPSP) that were not amenable to meta-analysis. DC = discriminate coefficient; RR = risk ratio; OR = odds ratio; SE = standard error; RTW = return to work

Category	Predictor	Outcome	Number of studies with a significant association	The effect size of the significant studies	Number of studies with a non-significant association	Comment
Sociodemographic factors	Age (years)	Leg pain 5 (n = 21,129)	3 (n = 20,915) Lagerbäck et al. [27] Shamji et al. [50] Weir et al. [59] Hareni et al. [65]	Beta (SE) = 0.02 (0.00) OR (95% CI) = 1.04 (1, 1.07) DC = 0.37 RR (95% CI) = 1 (1, 1)	2 (n = 214) Johansson et al. [41] Vucetic et al. [58]	Three out of five studies showed that increased age was significantly associated with persistent postoperative leg pain following lumbar microdiscectomy
		Pain not specified 6 (n = 883)	2 (n = 361) Moranjkic et al. [44] *Quon et al. [47] (age more than 50 years)	OR = 0.78 OR = 1.9 (1, 3.6)	5 (n = 813) Hegarty et al. [40] Den Boer et al. [36] Laufenberg-Feldmann et al. [62] Koen et al. [61] *Quon et al. [47]	Two out of six studies showed a significant association of age with postoperative pain following lumbar microdiscectomy. * Quon et al. categorized age as 34–40, 41–50, and >50 years; only age >50 years showed a significant association
	Gender	Leg pain 2 (20,565)	1 (n = 6,468) Lagerbäck et al. [27]	Beta (SE) = 0.32 (0.07)	1 (n = 14,097) Hareni et al. [65]	One out of two studies showed that gender was a significant predictor for persistent post-surgical leg pain in linear regression
		Pain not specified 5 (n = 607)	NA	NA	5 (n = 607) Hegarty et al. [40] Moranjkic et al. [44] Quon et al. [47] Laufenberg-Feldmann et al. [62] Koen et al. [61]	None of the five studies showed a significant association between age and pain
	Marital status and spousal support	Pain not specified 2 (n = 333)	(n = 42) Schade et al. [49]	Beta= -0.39; P= 0.01	(n= 291) Quon et al. [47]	One out of two studies showed a significant association with the persistent post-surgical pain-not specified
	Smoking	Leg pain 3 (n= 20,815)	1 (n = 6,468) Lagerbäck et al. [27]	Beta (SE) = 0.9 (0.08)	2 (n= 14347) Shamji et al. [50] Hareni et al. [65]	One out of the four studies showed smoking was significantly associated with post-surgical leg pain after the LDS
		Pain not specified			1 (n = 291) Quon et al. [47]	Smoking was not associated with pain
Medical factors	Body mass index (BMI)	Leg pain 2 (n = 6,843)	1 (n = 6468) Lagerbäck et al. [27]	Beta (SE) = 0.04 (0.01)	Siccoli et al. [52] (n = 552)	One out of the three studies showed that higher BMI was significantly associated with persistent post-surgical leg pain
		Pain not specified 2 (n = 142)	1 (n = 100) Laufenberg-Feldmann et al. [62]	Beta (95% CI) = 0.13 (.01–.25)	1 (n = 42) Schade et al. [49]	One out of the two studies showed BMI was significantly associated with persistent post-surgical leg pain
	Comorbidity	Leg pain 2 (n = 6,628)	2 (n= 6628) Lagerbäck et al. [27] Vucetic et al. [58]	Beta (SE) = 0.83 (0.2) OR (95% CI) = 2.5 (1.10, 5.4)		Two studies showed comorbidities were significantly associated with persistent post-surgical pain
	Axial joint pain/chronic pain 2 (n = 210)	Pain 1 (n = 110)	1 (n = 210) Voorhies et al. [57]	P = 0.004	1 (n = 100) Laufenberg-Feldmann et al. [62]	Preoperative axial or chronic pain was associated with persistent post-surgical leg pain
	Previous hospitalization	Leg pain 1 (n = 100)	1 (n = 100) Weir [59]	DC = 0.63		The author only reported association as a predictor of poor outcomes and did not report if the association was significant or not
	Genetic factor	Back pain 1 (n = 176)	Rut et al. [48] 1 (n = 176)	Beta (P) = -1.45 (0.003), -1.2 (0.046), -1.3 (0.014)	1 (n = 176) Rut et al. [48]	rs4633 allele T, rs4680 allele A, COMT haplotype L, respectively COMT haplotype L = non-significant
Disk herniation-related factors	Preoperative leg pain severity	Leg pain 6 (n = 7,830)	1 (n = 6,468) Lagerbäck et al. [27]	Beta (SE) = 0.11 (0.01)	5 (n = 1362) Den Boer et al. [36] Johansson et al. [41] Vucetic et al. [58] Shamji et al. [50] Udby et al. [66]	One out of six studies showed a significant association between higher preoperative leg pain intensity the persistent post-surgical leg pain
		Pain not specified	NA	NA	2 (n = 92) Ford et al. [39]	
	Preoperative low back pain severity	Leg pain 2 (n = 714)	NA	NA	2 (n = 714) Ford et al. [39] Udby et al. [66]	Higher preoperative LBP was not associated with persistent leg pain after the surgery
		Pain not specified 1 (n= 92)	1 (n = 92) Ford et al. [39]	Beta (95% CI) = -0.4 (-0.7, -0.1)	NA	Higher preoperative low back pain severity was associated with persistent post-surgical pain
	Preoperative pain, not specified	Pain not specified 7 (n = 927)	5 (555) Koen et al. [61] Laufenberg-Feldmann et al. [62] Moranjkic et al. [44] Quon et al. [47]	Beta (SE) = 0.01 (0.3) *Beta (95%CI) = 0.36 (0.61, 0.56) OR = 2.09 Pain intensity 8-9 OR = 1.80 (1, 3.1) Pain intensity 10 OR = 3.30 (1.6, 6.7)	4 (n = 663) Schade et al. [49] Den Boer et al. [36] *Quon et al. [47] Hegarty 2012(40)	Categorized pain as less than 7, 8-9, and 10; pain severity of 7 or less than 7 was not significantly associated with postoperative pain. !: Multivariate regression analysis *: Linear regression
	Preoperative neurological symptoms	Leg pain 3 (n = 444)	(n= 350) Weir [59] Shamji et al. [50]	Sensory abnormalities (DC = -0.19) OR (95% CI) = 24 (9, 73)	1 (n = 94) Ford et al. [39]	One out of the two studies showed that sensory findings were associated with a favorable outcome
		Pain not specified 5 (n = 717)	2 (n= 383) * Quon et al. [47] Ford et al. [39]	OR (95% CI) = 1.70 (1.1, 2.6)	4 (n= 680) Schade et al. [49] Quon et al. [47] Moranjkic et al. [44] Den Boer et al. [36]	Among all the studies, only abnormal reflexes were associated with postoperative pain. *: Only (absent reflexes) reflexes were a significant predictor of the PPSP, but other examination findings such as SLR, sensory findings, and muscle weakness were not
	Preoperative pain duration (not specified)	Leg pain 6 (n = 21,289)	4 (n = 21,037) Lagerbäck et al. [27] Weir [59] Hareni et al. [65]# Siccoli et al. [52]**	Beta (SE) = 0.04 (0.08) DC = 0.55 OR (95% CI) = 1.3 (1.2, 1.5) OR (95% CI) = 1.2 (1.1, 1.3) HR (95% CI) = 1.1 (1.1, 1.3)	3 (n = 14349) Ford et al. [39] *Vucetic et al. [58] Hareni et al. [65]	Four of the six studies showed a significant association between the longer pain duration of preoperative pain and persistent post-surgical leg pain. *: Vucetic et al. categorized pain as longer than seven months: less than seven months #: Categorized pain into 3–6 months, 6–12 months, 12–24 months, and more than 24 months **: Continuous variable
		Pain not specified 4 (n = 495)	2 (n = 361) Moranjkic et al. [44] Quon et al. [47]	OR = 0.94 *OR (95% CI) = 1.80 (1.1, 2.9)	3 (1 = 427) Ford et al. [39] Schade et al. [49] * Quon et al. [47] 6 weeks to 3 months, 3 to 6, months, and >6 months all	Two of the four studies showed that a longer duration of sciatica was associated with pain *: Duration of pain >12 weeks
	Duration of back pain	Leg pain 1 (n = 100)	1 (n = 100) Weir [59]	0.25		
	Preoperative disability score	Leg pain 2 (n = 714)	NA	NA	2 (n= 714) Ford et al. [39] Udby et al. [66]	Preoperative functional disability was not associated with the persistent postoperative pain leg and back pain after the surgery
		Pain not specified. 3 (n = 422)	1 (n= 53) Hegarty et al. [40]	Beta (SE)= 0.22 (0.11)	2 (n= 367) Den Boer et al. [36] Ford et al. [39]	One out of three studies showed that higher preoperative functional disability was associated with postoperative pain after the surgery.
	Time to surgery	Leg pain	Siccoli et al. [52]	HR (95% CI) = 0.68 (0.51, 0.9)	NA	
	Ruptured annulus	Leg pain	(n= 160) Vucetic et al. [58]	OR (95%CI) = 2.5 (1.20 (4.8)	NA	The ruptured annulus was significantly associated with persistent post-surgical leg and back pain.
	Preoperative pain management	Leg pain (n = 100)	(n= 100) Weir [59]	Physiotherapy (DC = 0.2) Preoperative analgesic use (DC = -0.18)	NA	Preoperative use of analgesics was a predictor of favorable outcomes, and physiotherapy was a predictor of unfavorable outcomes
	Previous injection therapy	Pain not specified (n = 298)	(n=298) Willems et al. [63]	OR (95% CI) = 2.02 (1.00, 4.07)		Previous injection therapy was a significant predictor of persistent postoperative pain after lumbar microdiscectomy
	The extent of neural compromise (Major vs. none)	Pain (n = 42)	(n= 42) Schade et al. [49]	SE= 0.30. P= 0.01	NA	The extent of neural compromise was significantly associated with PPSP.
	Number and level of the discectomy	Leg pain 1 (100)	Weir [59]	DC= 0.19	NA	Multiple levels of discectomy were predictors of poor outcomes
	Herniation type	Pain 2 (n = 78)	(n=36) Moranjkic et al. [44]	OR = 1.08	1 (n = 42) Schade et al. [49]	One out of two studies showed a significant association
Work-related	Compensation status	Leg pain 1 (n = 100)	1 (n = 100) Weir [59]	DC = 0.31	NA	Compensation status was a predictor of persistent post-surgical leg pain
		Pain not specified. 3 (n = 493)	3 (493) Quon et al. [47] Voorhies et al. [57] Ford et al. [39]	OR (95% CI) = 4.30 (2.4, 7.9) P = 0.00075 Beta (95% CI) = 2.20 (0.8, 3.6)	NA	Compensation status was significantly associated with PPSP
	Personal Injury	Pain	(n= 110) Voorhies et al. [57]	P= 0.0053	NA	Personal injury was a significant predictor of PPSP after the lumbar microdiscectomy
	Time off work before the surgery	Leg pain	Weir [59] (n = 42)	DC = -0.14	NA	Time off work was a predictor of a favorable outcome after the lumbar microdiscectomy
Psychological factors	Depression	Leg pain 1 (n = 67)	1 (n = 67) Chaichana et al. [35]	Beta = -0.06; P = 0.01		One of the two studies showed a significant association between depression and persistent post-surgical leg pain
		Pain Not specified. 3 (n = 386)	1 (n = 291) Quon et al. [47]	OR (95% CI) = 1.04 (1.02, 1.08)	2 (n = 95) Hegarty et al. [40] Schade et al. [49]	One out of the three studies showed depression was significantly associated with postoperative pain
	Anxiety	Pain not specified. 2 (n = 153)	NA	NA	2 (n = 153) Laufenberg-Feldmann et al. [62] Hegarty et al. [40]	Anxiety was not a significant predictor of PPSP after the lumbar microdiscectomy
	Psychological distress not specified	Leg pain	NA	NA	(n = 160) Vucetic et al. [58]	One out of the two studies showed a significant association with PPSP- leg pain and back pain
		Pain- not specified 1 (n = 110)	(n = 110) Voorhies et al. [57]	P = 0.022	NA	Higher psychological distress was associated with postoperative pain after lumbar microdiscectomy
	Somatization	Leg pain 1 (n = 67)	1 (n = 67) Chaichana et al. [35]	Beta = -0.14	NA	Somatization was a significant predictor of persistent post-surgical leg and back pain
	Patient’s preoperative expectations of RTW	Leg pain 1 (n = 55)	(n = 55) Johansson et al. [41]	OR (95% CI) = 8.20 (1.7, 41.1)	1 (n = 55) * Johansson et al. [41]	A low chance of RTW showed a significant association with PPSP leg pain *: High chances of RTW within three months postoperatively
	Negative outcome expectations	Pain 1 (n = 277)	Den Boer et al. [36] (n = 277)	SE = 0.11 P =< 0.001	NA	Negative outcome expectations were significantly associated with the PPSP after lumbar microdiscectomy

**Table 5:** Predictors for postoperative disability that were not amenable to meta-analysis.

Category	Predictor	Number of studies with a significant association	The effect size of significant studies	Number of studies with a non-significant association	Comment
Demographics and social factors	Age (years) 3 (n = 110 + 298)	2 (n = 36) + 298 *Moranjkic et al. [44] Willems et al. [63]	OR = 0.25 OR (95% CI) = 1.03 (1.00, 1.05)	1 (n = 40) Lee et al. [42]	Two out of two studies showed a significant association between age and functional disability *: Disability outcome was reported on the ordinal or binary scale *: Only OR was reported %= reported functional disability as a binary outcome and measured it with Ronald-Morris disability questionnaire (RMDQ)
	Gender 8 (n = 7,436)	1 (n = 6,531) Lagerbäck et al. [27]	Beta (SE) = 2.74 (0.42)	7 (n = 968) Moranjkic et al. [44] Shrestha et al. [51] Lee et al. [42] Den Boer et al. [36] Johansson et al. [41] Patel et al. [46] Solberg et al. [53]	One out of eight studies showed that the female sex was significantly associated with a postoperative disability after lumbar microdiscectomy
	Education 4 (n = 575)	Shrestha et al. [51] (n = 63)	Beta (SE) = 3.028 (1.330)	3 (n = 512) Johansson et al. [41] Solberg et al. [53] Den Boer et al. [36]	One out of the four studies showed that a lower level of education was significantly associated with functional disability. Patients with lower education had higher odds of functional disability
	Smoking 3 (n = 6,711)	2 (n = 6,531) Lagerbäck et al. [27] Shrestha et al. [51]	Beta (SE) = 6.20 (0.5) Beta (SE) = -4.302 (2.083)	1 (n = 180) Solberg et al. [53]	Two out of three studies showed that smoking was associated with a functional disability after lumbar microdiscectomy
Medical/Herniation-related factors	Comorbidity 3 (n = 6,690)	1 (n = 6,468) Lagerbäck et al. [27]	Beta (SE) = 0.33 (0.05)	2 (n = 222) Solberg et al. [53] Schade et al. [49]	One out of three studies showed that comorbidity was significantly associated with a disability after lumbar microdiscectomy
	Preoperative duration of symptoms sciatica 6 (n = 6,958)	3 (n = 6,633) Ford et al. [39] Moranjkic et al. [44] Lagerbäck et al. [27]	Beta (95% CI) = 3.30 (0.7, 5.9)! OR = 0.29 Beta (SE) = 3.79 (0.48) #	3 (n = 145) Lee et al. [42] Schade et al. [49] Shrestha et al. [51]	Two out of four studies showed that experiencing sciatica symptoms for a shorter duration was protective !: preoperative duration was on a continuous scale (months) #: duration >3 months (reference <3 months)
	Preoperative leg pain 6 (n = 1,521)	1 (n = 70) Moranjkic et al. [44] Ziegler et al. [64]	OR = 4.7002 Beta (SE) = 10.07 (1.63)	4 (n = 895) Lee et al. [42] Johansson et al. [41] Solberg et al. [53] Udby et al. [66]	One out of six studies showed that preoperative pain severity was significantly associated with postoperative functional disability
	Pain intensity back 3 (520)	1 (n = 298) Willems et al. [63]	OR (95% CI) = 1.01 (1.00, 1.02)	2 (n = 222) Lee et al. [42] Solberg et al. [53]	One out of three studies showed that preoperative back pain severity was associated with functional disability after the lumbar microdiscectomy %: reported functional disability as a binary outcome and measured it with the Ronald-Morris disability questionnaire (RMDQ)
	Leg pain is a predominant symptom	1 (n = 63) Shrestha et al. [51]	Beta (SE) = 15.567 (3.85)		As a predominant symptom, leg pain was significantly associated with functional disability
	Back pain is a predominant symptom	1 (n= 63) Shrestha et al. [51]	Beta (SE) = 9.64 (3.571)		
	Back pain and leg pain a dominant symptom	1 (n = 63) Shrestha et al. [51]	Beta (SE) = -12.213 (3.78)		
	Presence of neurological symptoms 5 (n = 547)	1 (n = 95) Ford et al. [39]	Beta = 18.40 95% CI = 5.9, 30.9)	4 (n = 452) Moranjkic et al. [44] Schade et al. [49] Den Boer et al. [36] Shrestha et al. [51]	One of the five studies showed that preoperative neurological symptoms were poor predictors of postoperative disability
	Preoperative disability 9 (n = 1,586)	3 (n = 105) Schade et al. [49] Shrestha et al. [51] Lagerbäck et al. [27]	Beta = 0.33, P = 0.001 Beta (SE) = -0.42 (0.979) Beta (SE) = 0.19 (0.01)	6 (n = 1,478) Den Boer et al. [36] Lee et al. [42] Solberg et al. [53] Udby et al. [66] En'Wezoh et al. [38] Willems et al. [63]	Two out of six studies showed that higher preoperative functional disability was significantly associated with poor postoperative functional disability %: reported functional disability as a binary outcome and measured it with the Ronald-Morris disability questionnaire (RMDQ)
	Herniation type (Extrusion/Sequestration vs. protrusion) 4 (n = 181)	Shrestha et al. [51] (n = 63)	Beta (SE) = -3.779 (1.72)	3 (n = 152) Lee et al. [42] Moranjkic et al. [44] Schade et al. [49]	One of the four studies showed a significant association of the herniation type (sequestration) with disability
	The extent of neural compromise (Major vs. none) 1 (n = 42)	1 (n = 42) Schade et al. [49]	Beta = -0.46	NA	The extent of neural compromise was significantly associated with postoperative functional disability
	Revision surgery 1 (n = 40)	Lee et al. [42] (n = 40)	OR (95% CI) = 36.45 (1.93, 689.57)	NA	A single study showed that revision surgery was associated with functional disability
	Preoperative medication use	1 (n= 298) Willems et al. [63]	OR (95% CI) = 1.99 (1.01, 3.94)	NA	Preoperative medications were significantly associated with functional disability
	Prior back surgery	1 (n = 298) Willems et al. [63]	OR (95%CI) = 2.80 (1.34, 5.88)	NA	Pre-existing back pain was significantly associated with functional disability
Work-related factors	Physical workload 2 (n = 168)	1 (n = 63) Shrestha et al. [51]	Beta (SE) = 6.107 (2.22)	1 (n = 55) Johansson et al. [41]	One out of two studies reported that heavy workload was significantly associated with postoperative disability
	Compensation status 1 (n = 95)	1 (n = 95) Ford et al. [39]	Beta (95% CI) = 10.5 (2.1, 18.91)	NA	Compensation status was significantly associated with postoperative functional disability
	Weeks of sick leave and rehabilitation (Preoperative) 1 (n = 180)	1 (n = 180) Solberg et al. [53]	Beta = 0.2, P = 0.026	NA	A longer duration of preoperative sick leave (weeks) and rehabilitation was significantly associated with postoperative functional disability
	Job-related resignation 1 (n = 42)	1 (n = 42) Schade et al. [49]	Beta = 0.40	NA	Job-related resignation was significantly associated with postoperative functional disability
Psychological	Depression 3 (n = 289)	1 (n = 67) Chaichana et al. [35]	Beta = -0.69; P = 0.001)	1 (n= 222) *Schade et al. [49] Solberg et al. [53]	One out of the two studies showed a significant association between depression and poor postoperative functional disability
	Expectation- negative outcome 3 (n = 512)	2 (n= 332) Den Boer et al. [36] Johansson et al. [41]*	SE = 0.08; P = <0.01 OR = 13.8 (2.2, 79.8)	1 (n = 180) Solberg et al. [53]	Two out of three studies showed a significant association between negative outcome expectancies and post-operative functional disability *: High expectations for RTW were not significantly associated with postoperative functional disability
	Somatization 1 (n = 67)	1 (n = 67) Chaichana et al. [35]	Beta (SE) = -1.23 (-1.69)	NA	Each 10-point increase is associated with 12.3 points less improvement in ODI
	Fear of movement 1 (n = 277)	1 (n = 277) Den Boer et al. [36]	SE = 0.02		One out of the two studies showed a significant association between fear of movement and functional limitations/disability
	Passive coping 2 (n = 332)	1 (n = 277) Den Boer et al. [36]	SE = 0.04	1 (n = 55) Johansson et al. [41]	One study out of the two studies showed a significant association between passive coping with functional limitations/disability

**Table 6:** Predictors of return to work that were not amenable to meta-analysis. $ = working capacity as an outcome; £ = an essential factor for future research; RTW = return to work; HR = hazard ratio; OR = odds ratio

Category	Predictor	Number of studies with a significant association	The effect size of significant studies	Number of studies with a non-significant association	Comment
Demographics and social factors	Age (older age) 6 (n = 2,120)	N = 160 Vucetic et al. [58]	OR (95% CI) = 3.1 (1.30, 7.5) # (increase age)	5 (n = 1,960) Den Boer et al. [36]$ Johansson et al. [41] O’Donnell et al. [45] Than et al. [55] *Ziegler et al. [60]	One out of six studies showed that an increase in age was significantly associated with failure to RTW *: Age = categorical variable #: OR for age >41 years (vs. >7 months)
	Education 4 (n = 702) (Lower education)	2 (n = 511) Vucetic et al. [58] Ziegler et al. [60]	OR (95% CI) = 3.3 (1.4, 8) HR (95% CI) = 3.36 (1.56, 8.43)	2 (n = 237) Den Boer et al. [36]$ Johansson et al. [41]	Two out of the four studies reported that lower education was associated with increased odds of failure to RTW
	Income 2 (n = 1637)	1 (n = 1,286) O’Donnell et al. [45]	OR (95% CI) = 1.01 (1, 1.02)	1 (n = 351) * Ziegler et al. [60]	One out of two studies showed higher incomes were associated with higher odds of RTW after the lumbar microdiscectomy *: categorical variable
Medical and LDH-related factors	Comorbidity 3 (n = 627)	1 (n = 160) Vucetic et al. [58]	OR (95% CI) = 7.1 (2.70, 18.4)!	2 (n = 467) Than et al. [55] Ziegler et al. [60]*	One out of four studies showed a significant association of comorbidity with no RTW following lumbar microdiscectomy. *: reported as ASA score !: no comorbidity (reference = positive comorbidity)
	Duration of symptoms 5 (n=525)	2 (n = 1,446) *Vucetic et al. [58] O’Donnell et al. [45]	OR (95% CI) = 3.8 (1.60, 9.2) # OR (95% CI) = 0.98 (0.97, 0.99)	3 (n = 506) Den Boer et al. [36] Schade et al. [49] * Ziegler et al. [60]	Two out of five studies showed more prolonged duration of sciatica symptoms was associated with decreased odds of RTW *: categorical variable #: OR >7 months (vs. <7 months)
	Previous spine surgery (reference: no previous surgery) 1(n = 160)	1 (n = 160) Vucetic et al. [58]	OR (95% CI) = 2.5 (1, 5.9)		Previous spine surgery showed significant association with no RTW
	Opioid use before surgery (reference: no opioid use before surgery)	1 (n = 1,286) O’Donnell et al. [45]	OR (95% CI) = 0.54 (0.39, 0.75) £		Preoperative opioid use was associated with decreased odds of RTW
Work-related factors	Physical workload 3 (n = 1,523)	1 (n= 182) Den Boer et al. [36]	OR = 1.19; P < 0.005	(n = 1341) *Johansson et al. [41] O’Donnell et al. [45]	One out of the three studies showed higher odds of failure to RTW with heavy work such as prolonged standing, carrying, twisting, and lifting *: categorized workload into heavy, moderate, and light work. All non-significant
	Preoperative work status 3 (n = 660)	2 (n= 478) Than et al. [55] Ziegler et al. [60]	OR (95% CI) = 76.61 (14.29, 410, 82)! HR (95% CI) = 0.96 (0.95, 0.97)	1(n = 182) Den Boer et al. [36]$	Two out of three studies showed that patients working preoperatively had higher odds of returning to work after the lumbar microdiscectomy !: working preoperatively (ref: not working preoperatively) *: Preoperative sick leave (longer duration of preoperative sick leave was associated with less probability of sustaining the work postoperatively)
	Preoperative disability and workers’ compensation benefits 3 (n = 1,764)	1 (n= 351) Ziegler et al. [60]	HR (95% CI) = 2.84 (1.44, 5.62)*	2 (n= 1413) O’Donnell et al. [45] Than et al. [55]	One out of three studies showed not receiving benefits was associated with a higher probability of RTW. *: Not receiving workers’ compensation (reference = receiving social benefits)
	Legal representation 1 (n = 1,286)	1 (n = 1c,286) O’Donnell et al. [45]	OR (95% CI) = 0.57 (0.44, 0.73)£		Preoperative legal representation was associated with lower odds of sustaining RTW (reference: no legal representation)
Preoperative functional disability	Preoperative disability score 4 (n = 660)	1 (n = 350) Ziegler et al. [60]	HR (95% CI) = 0.99 (0.15, 1)	3 (n = 310) Den Boer et al. [36] *Schade et al. [49] Than et al. [55]	One out of four studies showed higher preoperative functional disability was associated with less probability of returning to work after the lumbar microdiscectomy. *: Combined score for pain and disability
	Preoperative quality of life 3 (n = 520)	N = 350 Ziegler et al. [60]	HR (95% CI) = 1.02 (1.01, 1.03)	2 (n = 169) Schade et al. [49] Than et al. [55]	One out of three studies showed that higher preoperative quality was associated with a higher probability of sustaining RTW
Psychological	Depression 1 (n = 42)	1 (n = 42) * Schade et al. [49]	Beta = 0.37; P = 0.01		Preoperative depression was associated with a lower probability of RTW
	Psychological distress (not specified) 2 (n = 1,446)	1 (n = 1,286) O’Donnell et al. [45]	OR (95% CI) = 0.36 (0.14, 0.9) £	1 (n = 160) Vucetic et al. [58]	One out of the two studies showed comorbid psychiatric conditions were significantly associated with a lower probability of RTW
	Pain coping strategy 2 (n = 196)	1 (n = 182) Den Boer et al. [36]$	OR= 1.08	1 (n = 55) Johansson et al. [41]	One of the two studies showed passive pain coping strategies were associated with a lower probability of RTW
	Fear of movement/ re-injury 1 (n = 182)	1 (n = 182) Den Boer et al. [36]	OR = 1.09		One of the two studies showed that fear of movement or avoidance behavior was associated with a lower probability of RTW
	Low chance of a return to work within 3 months 2 (n = 347)	2 (n = 347) Johansson et al. [41] * Ziegler et al. [60]	OR (95% CI) = 19.50 (2.1, 179.2) HR (95% CI) = 3.49 (1.09, 11.15)		Low expectation of RTW was a significant predictor for no RTW, whereas moderate/some chances of RTW were not significant risk factors for RTW *: Similarly, the expectation for non-sick leave after surgery was associated with a higher probability of RTW [HR (95% CI) = 4.91 (1.45, 16.6)]
	Work-related stress. 2 (n = 212)	Schade et al. [49] (n=42)	Beta = 0.28, P = 0.01	1 (n =182) *Den Boer et al. [36]	Work-related stress was a significant factor for failure to RTW in one out of two studies

**Table 7:** Factors with consistent non-significant association with persistent postoperative pain (PPSP).

Category	Predictor	Outcome	Studies	Sample
Sociodemographic	Education	Leg pain	Vucetic et al. [58] (n = 160) Johansson et al. [41] (n = 55)	N = 215
		Pain not specified	Den Boer et al. [36] (n = 277)	N = 277
Preoperative functional status	Preoperative SF-physical component score/quality of life	Leg pain	Hareni et al. [65] Udby et al. [66]	N = 2,029
		Pain not specified	Hegarty et al. [40] Schade et al. [49]	N = 95
Work-related factors	Workload (heavy, moderate, and light work)	Leg pain	Johansson et al. [41]	N = 55
	Job-related resignation	Pain not specified	Schade et al. [49]	N = 42
	Occupational mental status	Pain not specified	Schade et al. [49]	N = 42
	Employment status (not working)	Pain not specified	Quon et al. [47]	N = 291
Disc-related factors	Degenerative changes	Leg pain	Udby et al. [66] Sørlie et al. [54]	N = 798
		Pain- not specified	Schade et al. [49]	N = 42
	Structural changes on MRI at the affected level of disc-herniation	Pain- Not specified	Willems et al. [63]	N = 298
Medical	Alcohol	Leg pain	Vucetic et al. [58]	N = 160
Psychological factors	Fear of movement	Leg pain	Den Boer et al. [36]	N = 277
		Pain not specified	Den Boer et al. [36]	N = 277
	Passive coping strategies	Pain not specified	Den Boer et al. [36] Johansson et al. [41]	N = 332
	Self-control	Pain not specified	Den Boer et al. [36]	N = 277
	Vitality	Pain not specified	Den Boer et al. [36]	N = 277

**Table 8:** Factors with a non-significant association for postoperative disability.

Category	Predictor	Studies	Number of studies (sample size)
Social factors	Drinking habits	Shrestha et al. [51]	1 (n = 63)
	Social support by a spouse	Schade et al. [49]	1 (n = 42)
Preoperative symptoms and disc-related factors	Disc degeneration	Schade et al. [49] Udby et al. [66]	2 (n = 662)
	Levels of disc herniation	Shrestha et al. [51]	1 (n = 63)
	Preoperative quality of life	Schade et al. [49] Udby et al. [66]	2 (n = 662)
	No Sitting activities	Willems et al. [63]	1 (n = 298)
	Preoperative pain (not specified)	Udby et al. [66]* Schade et al. [49] Den Boer et al. [36]	3 (n = 939) *Back pain
	Disc size (AP length), disc height, and Disc volume extracted	En'Wezoh et al. [38]	1 (n = 63)
Work-related factors	Occupational mental stress	Schade et al. [49]	1 (n = 42)
	Psychiatric conditions (not specified)	Solberg et al. [53]	1 (n = 180)
Medical factors	Body mass index	Schade et al. [49] Lagerbäck et al. [27] Solberg et al. [53]	3 (n = 6,690)
	Treatment before surgery (physiotherapy and medicine, physiotherapy, and medicine and epidural or nerve root block)	Shrestha et al. [51]	1 (n = 63)
Psychological factors	Self-control and vitality	Schade et al. [49]	1 (n = 42)

**Table 9:** Factors with non-significant association with failure to return to work.

Category	Predictor	Studies	Number of studies (sample size)
Social factors	Smoking	Than et al. [55] Ziegler et al. [60]	2 (n = 127)
	Ethnicity (Danish vs. immigrants)	Ziegler et al. [60]	1 (n = 329)
	Chronic alcoholism	Vucetic et al. [58]	1 (n = 160)
	Social support by a spouse	O’Donnell et al. [45] Schade et al. [49] Ziegler et al. [60]	3 (n = 1,679)
	Employer	Ziegler et al. [60]	1 (n = 329)
Medical factors	Body mass index	Schade et al. [49] Than et al. [55] Ziegler et al. [60]	3 (n = 520)
	Self-control Vitality	Schade et al. [49]	1 (n = 42)
	History of non-spinal surgeries	Vucetic et al. [58]	1 (n = 160)
	Preoperative treatments such as physiotherapy, chiropractic treatments, and psychotherapy use	Den Boer et al. [36] O’Donnell et al. [45]	2 (n = 1427)
Preoperative symptoms and disc-related factors	Preoperative pain	Den Boer et al. [36] Johansson et al. [41] Vucetic et al. [58] Ziegler et al. [60]	4 (n = 707)
	Leg pain intensity higher than back pain intensity	Ziegler et al. [60]	1 (n = 329)
	Preoperative examination findings/neurological symptoms	Den Boer et al. [36] Schade et al. [49] Vucetic et al. [58] Ziegler et al. [60]	4 (n = 672)
	Disc degeneration	Schade et al. [49]	1 (n = 42)
	The extent of the disc herniation (major vs. none)	Schade et al. [49]	1 (n = 42)
	Surgical type (microendoscopic vs. open discectomy)	Ziegler et al. [60]	1 (n = 351)
	Extent neural compromise	Schade et al. [49]	1 (n = 42)
	Surgical complications	Ziegler et al. [60]	1 (n = 329)
Work-related factors	Job satisfaction	Den Boer et al. [36]	1 (n = 182)
	Duration of sick leave	Den Boer et al. [36]	1 (n = 182)

Discussion

We found moderate certainty evidence that the female sex was probably associated with a small increased risk (2%) of persistent post-surgical leg pain and a large increased risk (11%) of failure to RTW after microdiscectomy for sciatica. Moderate certainty evidence also showed that older age was probably associated with a small increased risk for persistent disability after decompression surgery. Studies have tested approximately 50 predictors that could not be pooled, of which opioid use before surgery and legal representation at the time of surgery warrant additional investigation.

The key strengths of our review are that methodologically, our review was more rigorous as we accounted for non-significant variables and imputed 1 for excluded variables due to a non-significant association in the univariable analysis. Carrying only significant predictors to the multivariable analysis increases the risk of overestimation in the final analysis model. We presented our results with an absolute measure of association, such as risk difference. Compared to the relative measure of association, such as OR, and RR, the absolute measure of association, is essential to guide clinical decision-making. We performed subgroup analysis based on the risk of bias and further assessed the credibility of subgroup effects using the ICEMAN criteria.

Our review also suffered from a few limitations. Many predictors were only reported by a single study, due to which we could not perform a meta-analysis. Another limiting factor that precluded us from a meta-analysis of most variables was incomplete data reporting, such as many studies only reported p-values [38,46,57] or SE [36] or OR without 95% CI [44] or only beta-coefficient [49,53].

Compared to the previous systematic reviews, we identified more studies that previous reviews did not include [14,15,17,21]. Den Boer et al. [17] included 13 out of 15 (>85%) studies that reported composite scores. Our rationale for excluding studies with composite scores such as patient satisfaction and medication use was because composite scores can obscure the vital information specific to outcomes [67,68]. Composite scores are more useful when the outcome is rare, and combining multiple outcomes such as pain, disability, work capacity, doctor visits, analgesic use, sleep disturbances, patient’s opinion, or clinical examination [69] can reduce the type I error, but combining variables reduces meaningful information and makes the interpretation difficult [67,69].

Furthermore, den Boer et al. [17] included studies that analyzed outcomes data with unadjusted analysis and reported the positive and negative association based on the number of studies reporting a variable. Analyzing the association of baseline variables with the outcome in a multivariable-adjusted analysis accounts for the effect of potential known variables that can affect the outcome. Previous reviews included heterogeneous study designs such as RCT [21] and studies that analyze the association of various baseline variables with the outcomes in unadjusted analyses [14,15,17].

Interim of conducting our systematic review, potential new studies were published. Mehendiratta et al. [70] reported a significant association of younger age, males, and non-smokers, with symptom duration fewer than six weeks, and with disc herniation at L3 to L4 with a postoperative disability after the lumbar microdiscectomy. The study analyzed the predictive association of baseline variables in unadjusted analysis with postoperative disability. The adjusted multivariable analysis allows us to account for the effect modification and relationship between various baseline risk factors. Future studies should analyze the association of various baseline variables with postoperative pain, disability, and RTW in large sample-size studies and optimally adjusted models.

## Conclusions

Our review found moderate certainty evidence that the female sex had a higher probability of persistent leg pain and failure to RTW after a microdiscectomy for sciatica and that older age is probably associated with greater post-surgical impairment. We also identified the limitations in the current published literature such as heterogeneous reporting of the results, small study samples, and not consistently adjusting final models for important variables such as age, sex, and preoperative sciatica pain severity, which have shown significant association with postoperative outcomes after lumbar microdiscectomy. We also identified two important variables such as legal representation and preoperative opioid use that were not amenable to pooling but met our criteria for potential variables that may have a significant association with postoperative outcomes after lumbar microdiscectomy. Future research should explore the association between legal representation, preoperative opioid use, and persistent pain and impairment after microdiscectomy for sciatica in a large sample and methodologically rigorous studies.

## Appendices

**Table 10:** Evaluating the credibility of the subgroup effect of lack of optimal adjusted model based on the ICEMAN criteria.

Outcome: Failure to return to work (RTW), Predictor: Female sex (reference: male)					
Credibility criteria with answers					Comments
Is the analysis of effect modification based on comparison within rather than between trials?					
[X] Completely between	Mostly between or unclear	Mostly within	Completely within	-	Subgroup analysis was based on studies
For within-trial comparisons, is the effect modification similar from trial to trial?					
[×] Not applicable - No or one within-study comparison	Definitely no	Probably not similar or unclear	Mostly similar	Definitely similar	NA
For between-trial comparisons, is the number of trials large?					
[X] Very small	Rather small or unclear	Rather large	Large	-	The high-risk-of-bias group had two studies, and the group with low risk of bias had three studies
Was the direction of effect modification correctly hypothesized a priori?					
Definitely no	Probably no or unclear	Probably yes	[X] Definitely yes	-	We specified studies with a high risk of bias are likely to have a larger effect size
Does a test for interaction suggest that chance is an unlikely explanation of the apparent effect modification? (Consider irrespective of the number of effect modifiers)					
Chance a very likely	[X] Chance a likely explanation or unclear	The chance may not explain	Chance an unlikely	-	The test of interaction showed significant subgroup effects (P = 0.03)
Did the authors test only a small number of effect modifiers or consider the number in their statistical analysis?					
Definitely no	Probably no or unclear	Probably yes	[X] Definitely yes	-	Only tested subgroup analysis based on the risk of bias
Did the authors use a random-effects model?					
Definitely no	Probably no or unclear	Probably yes	[X] Definitely yes	-	Yes
If the effect modifier is a continuous variable, were arbitrary cut-points avoided?					
[X] Not applicable	Definitely no	Probably no or unclear	Probably yes	[] Definitely yes	
Very low credibility	Low credibility		Moderate credibility X		High credibility

Literature search strategy

Search strategy: OVID Medline

--------------------------------------------------------------------------------

1     exp Sciatic Neuropathy/su

2     Lumbar Vertebrae/su

3     ((surgery or surgical) adj3 (lumbar or sciatica)).mp.

4     or/1-3

Annotation: lumbar surgery

5     exp Diskectomy/

6     Radiculopathy/su

7     Decompression, Surgical/

8     Intervertebral Disc/su

9     Intervertebral Disc Displacement/su

10     (discectom* or microdiscectom* or diskectom* or microdiskectom*).mp.

11     or/5-10

Annotation: discectomy

12     Lumbar Vertebrae/ or lumbar.mp.

13     11 and 12

Annotation: lumbar discectomy

14     4 or 13

Annotation: lumbar surgery concept

15     prognosis/

16     exp risk/

17     exp PROBABILITY

18     exp Regression Analysis/

19     "analysis of variance"/ or multivariate analysis/

20     exp Epidemiologic Studies/

21     (discectom* or microdiscectom* or diskectom* or microdiskectom*).mp.

22     ((univariate or covariate or variance or covariance or multivariate or regression or adjusted or unadjusted or logistic or diagnostic) adj2 (analys* or model*)).mp.

23     (logistic adj2 regress*).mp.

24     ((cohort or observational) adj3 (study or studies or analy*)).mp. [mp=title, abstract, original title, name of substance word, subject heading word, floating sub-heading word, keyword heading word, organism supplementary concept word, protocol supplementary concept word, rare disease supplementary concept word, unique identifier, synonyms]

25     (longitudinal or retrospective or cross sectional or prospective).mp. [mp=title, abstract, original title, name of substance word, subject heading word, floating sub-heading word, keyword heading word, organism supplementary concept word, protocol supplementary concept word, rare disease supplementary concept word, unique identifier, synonyms]

26     (Follow up adj (study or studies)).tw.

27     questionnaire$.mp. or Questionnaires/

28     ep.fs.

29     or/15-28

30     14 and 29

31     limit 30 to yr="2017 -Current"

Database: Embase search strategy

--------------------------------------------------------------------------------

1     exp sciatic neuropathy/su [Surgery]

2     lumbar vertebra/su [Surgery]

3     ((surgery or surgical) adj3 (lumbar or sciatica)).mp. [mp=title, abstract, heading word, drug trade name, original title, device manufacturer, drug manufacturer, device trade name, keyword, floating subheading word, candidate term word]

4     lumbar disk hernia/su [Surgery]

5     1 or 2 or 3 or 4

6     exp discectomy/

7     exp radiculopathy/su [Surgery]

8     decompression surgery/ or nerve decompression/ or spinal cord decompression/

9     exp intervertebral disk/su [Surgery]

10     intervertebral disk hernia/su [Surgery]

11     (discectom* or microdiscectom* or diskectom* or microdiskectom*).mp.

12     or/6-11

13     exp lumbar spine/ or lumbar.mp.

14     12 and 13

15     5 or 14

16     cohort analysis/

17     probability/

18     trend study/

19     epidemiology/ or pharmacoepidemiology/

20     sensitivity analysis/

21     prognosis/

22     risk/

23     risk assessment/

24     risk factor/

25     exp regression analysis/

26     "analysis of variance"/

27     multivariate analysis/

28     (prognosis or prognostic or predict* or risk*).tw.

29     ((univariate or covariate or variance or covariance or multivariate or regression or adjusted or unadjusted or logistic or diagnostic) adj2 (analys* or model*)).tw.

30     (logistic adj2 regress*).tw.

31     ((cohort or observational) adj3 (study or studies or analy*)).tw.

32     (longitudinal or retrospective or cross sectional or prospective).tw.

33     (Follow up adj (study or studies)).tw.

34     questionnaire*.mp. or questionnaire/

35     (prevalen* or inciden* or pharmacoepidemiol*).tw.

36     ep.fs.

37     or/16-36

38     15 and 37

39     limit 38 to yr="2017 -Current"

Database: CINAHL (Ebsco) search strategy

--------------------------------------------------------------------------------

S31 S14 AND S30

S30 S15 OR S16 OR S17 OR S18 OR S19 OR S20 OR S21 OR S22 OR S23 OR S24 OR S25 OR S26 OR S27 OR S28 OR S29

S29 "questionnaire*"

S28 (MH "Questionnaires")

S27 Follow up N1 (study or studies))

S26 longitudinal or retrospective or cross sectional or prospective

S25 ((cohort or observational) N3 (study or studies or analy*))

S24 logistic N2 regress*

S23 ((univariate or covariate or variance or covariance or multivariate or regression or adjusted or unadjusted or logistic or diagnostic) N2 (analys* or model*)).

S22 discectom* or microdiscectom* or diskectom* or microdiskectom*

S21 (MH "Epidemiology+")

S20 (MH "Epidemiological Research")

S19 (MH "Analysis of Variance+")

S18 (MH "Regression+")

S17 (MH "Probability")

S16 (MH "Risk Assessment")

S15 (MH "Prognosis")

S14 S4 OR S13

S13 S11 AND S12

S12 "lumbar*"

S11 S5 OR S6 OR S7 OR S8 OR S9 OR S10

S10 discectom* or microdiscectom* or diskectom* or microdiskectom*

S9 (MH "Intervertebral Disk Displacement/SU")

S8 (MH "Intervertebral Disk+/SU")

S7 (MH "Decompression, Surgical")

S6 (MH "Radiculopathy/SU")

S5 (MH "Diskectomy")

S4 S1 OR S2 OR S3

S3 ((surgery or surgical) N3 (lumbar or sciatica))

S2 (MH "Lumbar Vertebrae/SU")

S1 (MH "Sciatica/SU")
